# From paroxysmal hemicrania to SUNCT: a unique presentation of herpetic zoster ophthalmicus: a case report

**DOI:** 10.3389/fopht.2025.1628665

**Published:** 2025-12-16

**Authors:** Po-Yu Tsai, Hui-Chen Su, Yu-Ming Chang

**Affiliations:** 1 Department of Neurology, National Cheng Kung University Hospital, College of Medicine, National Cheng Kung University, Tainan, Taiwan; 2 Institute of Allied Health Sciences, College of Medicine, National Cheng Kung University, Tainan, Taiwan

**Keywords:** headache, TACs, trigeminal autonomic cephalalgias, VZV infection, herpetic zoster ophthalmicus

## Abstract

**Background:**

Trigeminal autonomic cephalalgias (TACs) are characterized by unilateral headache with cranial autonomic symptoms. Sudden subtype changes may suggest secondary causes.

**Case presentation:**

A 66-year-old woman presented with a rapid shift from paroxysmal hemicrania to SUNCT. Varicella zoster virus (VZV) reactivation was confirmed by CSF analysis and aqueous humor PCR. Ocular involvement included panuveitis and papillitis. Symptoms resolved after 14 days of intravenous acyclovir.

**Conclusion:**

Rapid TACs subtype transformation should prompt evaluation for secondary causes. Early diagnosis of VZV can lead to favorable outcomes.

## Introduction

Trigeminal autonomic cephalalgias (TACs) are a group of primary headache disorders characterized by unilateral trigeminal distribution pain accompanied by prominent ipsilateral cranial autonomic symptoms, including conjunctival injection, lacrimation, rhinorrhea, or ptosis. The TAC spectrum includes cluster headache (CH), paroxysmal hemicrania (PH), short-lasting unilateral neuralgiform headache attacks with conjunctival injection and tearing (SUNCT), short-lasting unilateral neuralgiform headache attacks with cranial autonomic symptoms (SUNA), and hemicrania continua (HC). These entities are distinguished primarily by attack duration, frequency, and therapeutic response profiles. (1).

Secondary causes of TACs-like syndrome include intracranial tumors, vascular abnormalities, and intracranial infections (2). Varicella-zoster virus (VZV) reactivation has been implicated in TACs-like presentation, occurring either before or after rash development. However, previous reports had described headaches corresponding to a single, specific TACs subtype without documenting a transition between subtypes. (3–5). We report a case whose headache pattern evolved from PH-like to SUNCT-like presentation during early VZV reactivation, later complicated by herpes zoster ophthalmicus.

## Case presentation

A 66-year-old woman without history of headaches presented to our facility with a severe right-sided headache that progressively worsened in frequency and severity four days. Initially, she experienced continuous rhinorrhea from her right nostril, followed by throbbing, sharp pain extending from her right nostril to the right periorbital and temporal area. The highest visual analog scale (VAS) was 5. The attacks occurred 10–20 times daily, each lasting about 30 minutes with a brief interval. Over time, ipsilateral tearing and mild eyelid drooping developed subsequently.

**Figure 1:**
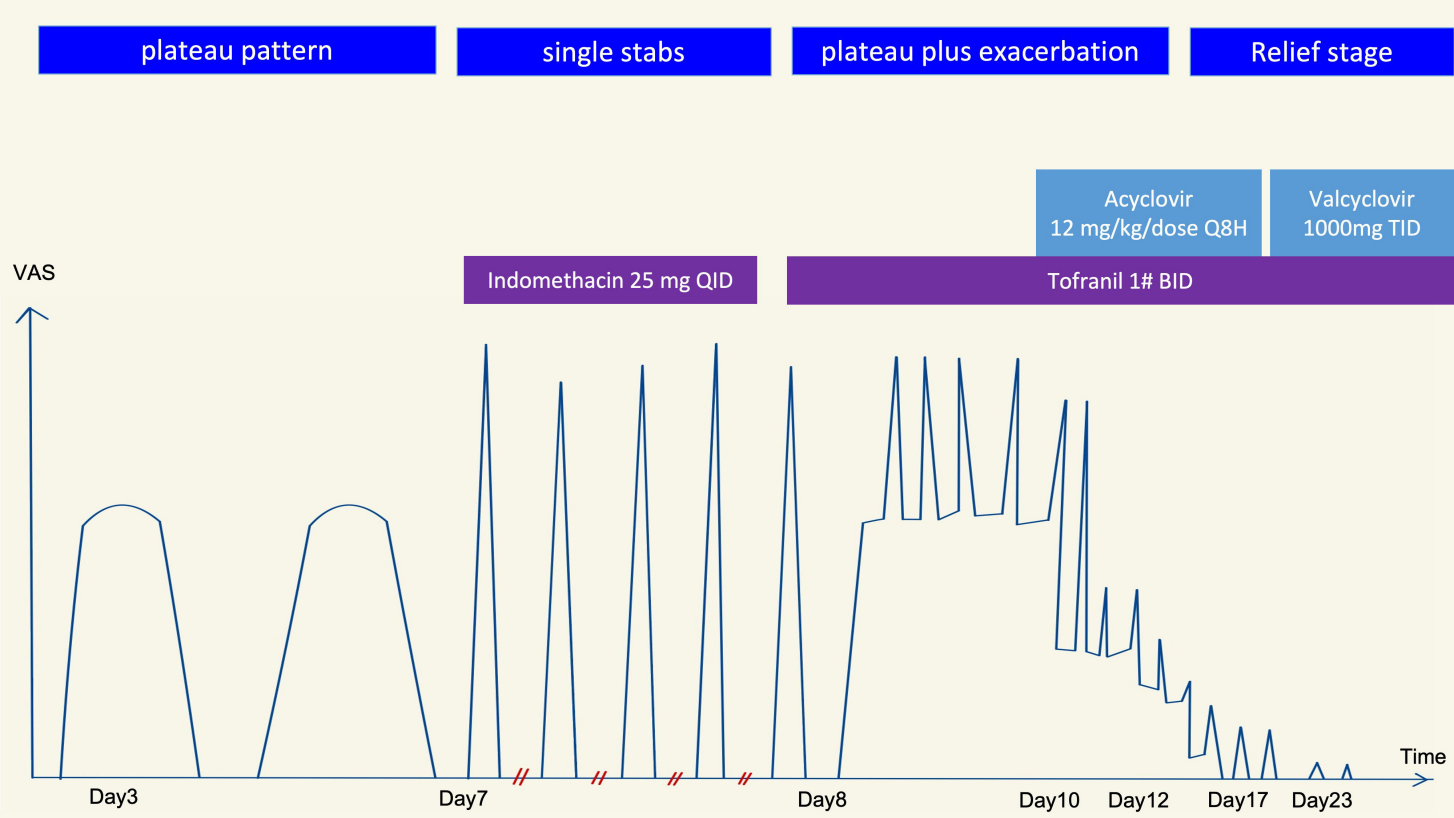
The evolution of the headache pattern of our patient since the headache onset. Initially, the headache had a ‘‘plateau pattern’’ with a duration and interval consistent with the typical presentation of PH. Indomethacin was ineffective. Within the same day, the headache transitioned from a ‘‘single stabbing pattern’’ to a SUNCT pattern. Finally, a ‘‘plateau plus exacerbation pattern’’ evolved, and there was no refractory period between each exacerbation. The headache rapidly improved following administration of intravenous acyclovir. PH, paroxysmal hemicrania; SUNCT, short-lasting unilateral neuralgiform headache attacks with conjunctival injection and tearing; VAS, visual analogue scale.

By day 7, her headache intensified (VAS 10) with increasing frequency up to 100 short attacks per day, lasting a few seconds each with intervals of less than five minutes. New autonomic features including periorbital redness and conjunctival injection appeared. On day 8, the headache worsened with prominent right-side lacrimation, rhinorrhea, drooping eyelid, conjunctival injection, and photophobia in the right eye. The temporal tingling, stabbing pain persisted and showed fluctuating peaks. There was no fever, nausea, vomiting, or neck stiffness, and the pain was unaffected by the Valsalva maneuver or postural changes. According to third edition of the International Classification of Headache Disorders (1), the headache initially resembled PH pattern but transitioned to a SUNCT-like pattern on day 8. **Figure 1** illustrates the headache pattern evolution throughout the disease course.

Physical examinations revealed right periorbital redness, and swelling with conjunctival injection. Neurological examinations revealed right partial ptosis along with binocular vertical diplopia on downward gaze which suggested potential sympathetic nerve dysfunction and right supranuclear nerve palsy, respectively. Of significant importance was the presence of hyperalgesia over the distribution of the right trigeminal nerve. Notably, tactile or pain stimulus did not elicit facial pain or exacerbate the headache, and meningeal signs were negative. No audible bruits were detected over the supraorbital, orbital, and posterior auricular regions, nor the pterion. Routine laboratory results were unremarkable. Cerebrospinal fluid (CSF) was clear with normal intracranial pressure, normal glucose and protein level. On day 10, the CSF Filmarray meningitis/encephalitis panel detected the presence of VZV, subsequently confirmed by quantitative PCR for VZV. Brain magnetic resonance imaging (MRI) showed no abnormalities.

On day 9, oral indomethacin 75 mg/day was administered, but was ineffective while imipramine 10 mg/day provided partial relief. Subsequently, intravenous acyclovir (12 mg/kg/day for 14 days) was administrated with topical acyclovir ointment given for ocular prophylaxis. Headache severity decreased within 48 hours, and autonomic symptoms gradually subsided.

Despite of partial symptoms improvement, right conjunctival injection and blurred vision persisted with a reduced visual acuity from 0.63 to 0.2. Ophthalmologic examinations revealed right eye chemosis, anterior chamber cells, vitritis and optic disc edema. PCR of the aqueous humor confirmed VZV DNA, consistent with VZV-related panuveitis with papillitis. Topical 1% prednisolone acetate eyedrops was prescribed.

**Figure 2:**
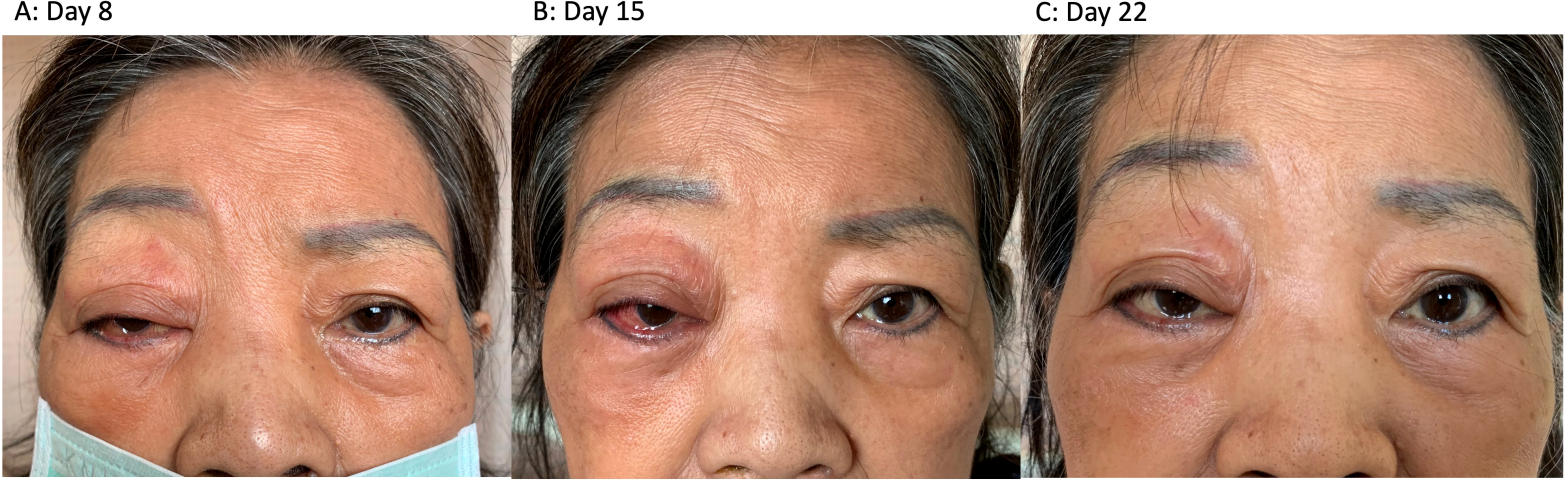
The evolution of facial symptoms **(A)** Day 8: The patient presented with periorbital redness, conjunctival injection, and droopy eyelid. **(B)** Day 15: After acyclovir administration on day 10, there was a partial improvement in the droopy eyelid. However, there was a persistent right conjunctival injection accompanied by blurred vision. **(C)** Day 22: Periorbital redness, swelling, and conjunctival injection significantly improved, and there was only mild residual ptosis. Throughout the course of the disease, the patient did not develop any vesicles around trigeminal nerve distribution.

After completion of intravenous acyclovir treatment, lacrimation and rhinorrhea, diplopia and periorbital inflammation resolved. (**Figure 2**). However, mild ptosis and hypoesthesia over the ophthalmic nerve distribution remained, with minimal intermittent stabbing pain with VAS 1. She was discharged on oral valacyclovir (1000 mg three times a day) At one-month follow-up, only mild intermittent tingling along the right nasal sidewall persisted. Continuous valaciclovir and topical steroid eyedrops led to the resolution of vitritis and papillitis.

## Discussion and conclusion

This case demonstrates a rapid transformation between TACs subtypes within two weeks. The dynamic evolution from PH to SUNCT highlights the heterogeneous activation of trigeminal-autonomic pathway during VZV reactivation. Additionally, the presence of erythematous skin changes, localized swelling, and indomethacin resistance suggested an underlying secondary etiology. Early recognition of VZV reactivation and prompt antiviral therapy led to a favorable outcome.

The parasympathetic fiber originating from the superior salivary nucleus and projecting to the sphenopalatine ganglion was important for the cranial parasympathetic autonomic features such as rhinorrhea and lacrimation (6). VZV is latent in various ganglia, including those of cranial nerves, dorsal roots, and autonomic ganglia of the enteric, sympathetic, and parasympathetic nervous systems (7). Although cases of VZV-related autonomic dysfunction are documented, the majority of them manifest as dorsal root ganglionopathy, predominantly impacting the gastrointestinal and urogenital systems (8). Involvement of enteric neuron may present with acute colonic pseudo-obstruction and involvement of sacral plexus may result in urinary retention, impotence, or loss of anal and bulbocavernosus reflexes (9–12). An *in vitro* analysis of sensory and autonomic ganglia (superior cervical, nodose, sphenopalatine, ciliary, otic ganglion) from cadavers suggesting the latency of the VZV genome in ganglia other than the trigeminal ganglion (13). Cranial nerve dysautonomia caused by VZV is limited, but a nociceptive stimulation after VZV reactivation could activate parasympathetic reaction through trigeminal autonomic reflex by stimulating trigeminal nerve, resulting in presentations similar to TACs (14, 15). The rapid transformation from a PH-like to a SUNCT-like pattern in our patient may reflect transient dysfunction across both trigeminovascular and parasympathetic pathways. Activation of the trigeminovascular system during VZV reactivation could sensitize second-order neurons in the trigeminal nucleus caudalis, facilitating cross-talk with parasympathetic outflow through the superior salivatory nucleus and sphenopalatine ganglion (16). Such neural interplay might transiently alter the threshold and periodicity of autonomic activation, explaining the abrupt shift in attack duration and frequency. Central sensitization may further amplify this effect, producing overlapping or evolving headache phenotypes within a short time span (17).

In our case, the right facial hyperalgesia and headache with binocular vertical diplopia indicated cranial nerve dysfunction involving the trigeminal nerve and supranuclear nerve. However, the simultaneous presentation of ipsilateral rhinorrhea and lacrimation highly suggested that the VZV might further have reactivated at the sphenopalatine ganglion and superior cervical ganglion leading to autonomic dysfunction. Previous reports have described VZV-associated TACs including PH, SUNCT, and HC subtypes. (3–5, 18–24). Among these, SUNCT is the most frequently reported subtype (2, 3). Notably, most cases describe TACs after VZV reactivation; in contrast, our case is distinctive in showing subtype transformation preceding overt ophthalmic zoster, underscoring a potential early manifestation of viral reactivation. (3). To our knowledge, the phenomenon of a rapid shift or transition between different TAC subtypes has not been previously reported.

Our case has unique presentations, including prominent autonomic symptoms with rhinorrhea at onset and a rapid transition from a PH-like headache to a SUNCT-like pattern. Such dynamic evolution of TAC subtypes has not been previously reported in association with VZV infection. This observation suggests that VZV-related TACs may manifest with not only diverse clinical phenotypes but also subtype transitions during the disease course. In addition, conjunctival injection and tearing mimicking SUNCT-like presentation caused by VZV infection might have masked the early detection of VZV-related panuveitis with papillitis. Although the disease course didn’t exhibit typical vesicle eruption and was characterized only by periorbital redness, it is plausible that the cutaneous manifestations were concealed during the reactivation of the virus in the autonomic neurons which didn’t project to the skin. This single-case observation should be interpreted with caution, as neuroimaging did not reveal definitive ganglion involvement, and clinical improvement may have resulted from the synergistic effects of antiviral and anti-inflammatory therapy.

In summary, we presented a rare case of TACs-like syndrome preceding a VZV reactivation, accompanied by severe ocular involvement resulting in visual acuity impairment. This case underscores the importance of recognizing atypical features in secondary TACs including onset at an older age, abnormalities in general or neurological examination, attack-related features not fulfilling ICHD-3 criteria, and refractoriness to conventional treatment. Our case underscores the importance of closely monitoring rapid changes in headache patterns, offering valuable insights into the atypical features of TACs and atypical presentation of VZV infection in clinical practice. Early detection of the underlying causes and appropriate treatment of atypical TACs are crucial for achieving favorable outcomes.

## Data Availability

The original contributions presented in the study are included in the article/supplementary material. Further inquiries can be directed to the corresponding authors.
